# Mathematical Model for Length Control by the Timing of Substrate Switching in the Type III Secretion System

**DOI:** 10.1371/journal.pcbi.1004851

**Published:** 2016-04-14

**Authors:** Maulik K. Nariya, Johnny Israeli, Jack J. Shi, Eric J. Deeds

**Affiliations:** 1 Department of Physics and Astronomy, University of Kansas, Lawrence, Kansas, United States of America; 2 Center for Computational Biology, University of Kansas, Lawrence, Kansas, United States of America; 3 Department of Molecular Biosciences, University of Kansas, Lawrence, Kansas, United States of America; 4 Sante Fe Institute, Santa Fe, New Mexico, United States of America; Colorado State University, UNITED STATES

## Abstract

Type III Secretion Systems (T3SS) are complex bacterial structures that provide gram-negative pathogens with a unique virulence mechanism whereby they grow a needle-like structure in order to inject bacterial effector proteins into the cytoplasm of a host cell. Numerous experiments have been performed to understand the structural details of this nanomachine during the past decade. Despite the concerted efforts of molecular and structural biologists, several crucial aspects of the assembly of this structure, such as the regulation of the length of the needle itself, remain unclear. In this work, we used a combination of mathematical and computational techniques to better understand length control based on the timing of substrate switching, which is a possible mechanism for how bacteria ensure that the T3SS needles are neither too short nor too long. In particular, we predicted the form of the needle length distribution based on this mechanism, and found excellent agreement with available experimental data from *Salmonella typhimurium* with only a single free parameter. Although our findings provide preliminary evidence in support of the substrate switching model, they also make a set of quantitative predictions that, if tested experimentally, would assist in efforts to unambiguously characterize the regulatory mechanisms that control the growth of this crucial virulence factor.

## Author Summary

The Type III Secretion System (T3SS) is a molecular needle that allows pathogenic bacteria (e.g. *Salmonella*) to inject proteins into host cells and control their behavior. Two mechanisms have been proposed to explain how bacteria regulate the length of the T3SS needle, but to date neither of these mechanisms has been subjected to any rigorous quantitative analysis. In this work we constructed a mathematical model for one of these mechanisms, namely length control via the timing of substrate switching. We showed that this model is quantitatively consistent with experimental data from *S. typhimurium.* In addition to providing evidence for the substrate switching mechanism, our work provides a framework for future quantitative evaluation of length control in the T3SS.

## Introduction

The Type III Secretion System (T3SS) is a major virulence factor found in a large number of pathogenic bacteria, including prominent disease-causing organisms such as *Salmonella typhimurium*, *Shigella spp.* and *Yersinia pestis* [1]. The structure of this massive secretion machinery is homologous to that of the bacterial flagellum: it consists of a base complex that spans the inner and outer membranes of gram-negative bacteria and a long needle that protrudes into the surrounding environment [1–4]. A specific set of “tip” and “translocon” proteins at the end of the needle can form a pore in the plasma membranes of cells from the host organism (e.g. epithelial cells lining the large intestines in mammals), creating a narrow channel from the bacterial cytoplasm to that of the eukaryotic cell [1, 5–8]. The bacterium uses this channel to inject various “effector proteins” into the host cell; these proteins alter the behavior of the cell, in some cases leading to endocytosis of the bacterium and eventual invasion of the epithelial layer [1, 4]. Correct functioning of this “injectisome” apparatus is crucial for pathogenesis in many bacterial species, making the T3SS an attractive target for the development of antimicrobials and vaccines [1, 9, 10].

The structure of the T3SS system has been studied in detail, resulting in near-atomic resolution models of the base complex, the needle itself and the interaction of the needle with tip and translocon proteins [2–8, 11, 12]. Since functioning needle complexes are required for efficient pathogenesis, the assembly of this complex structure is highly regulated. One parameter of particular importance is the length of the needle: if the needle is too short, it will not emerge past the lipopolysaccharide (LPS) layer on the outer membrane and thus will not be able to engage eukaryotic cells. On the other hand, if the needle is too long, the efficiency of transport/injection may be reduced, and long needles can also fracture due to shear stress, which could render the T3SS inactive [1, 2, 5]. Fairly precise control of needle length is thus essential to injectisome function. The needle itself consists of a polymer of “needle” proteins, called PrgI in *Salmonella* and YscF in *Yersinia*; these proteins form a helical structure with a ∼ 25 Å pore in the middle [5–7]. The needle is assembled via export of these subunits from the base: as subunits are exported, they are incorporated into the end of the needle and the needle becomes longer. When the needle reaches approximately the correct length, this information is somehow relayed to the base complex, which ceases secreting needle proteins and begins to secrete the tip proteins and other factors needed for virulence and invasion [1].

Two mechanisms for controlling the length of the needle have been proposed. Cornelis and co-workers have posited that a dedicated protein might serve as a “molecular ruler.” The idea in this case is that the C-terminus of the ruler protein can interact with the base, while the N-terminus can interact with the tip of the growing needle. As a result of this interaction, the length of the ruler is then compared to the length of the needle, and when the ruler protein is stretched to its full length (say, adopting a fully extended *α*-helical conformation), this information is relayed to the base. At that point the base begins secreting tip proteins and the needle complex becomes mature [1]. In *Yersinia spp.* and related organisms, the putative ruler protein has been identified as YscP: it has been shown that lengthening this protein by inserting amino acid sequences resulted in longer needles, while deletions that shortened the protein shortened the needles, consistent with the expected behavior of a molecular ruler [13, 14]. Ruler proteins have since been found to be involved in the length control of other structures, including the bacterial flagellum [1, 15–18].

In *Salmonella* and *Shigella*, however, there is an “inner rod” complex that is distinct from the outer needle complex; this inner rod spans the inner and outer membranes inside the base complex and is composed of a different protein from the needle itself [1, 2]. The existence of this inner rod led to the proposal of an alternative model for needle length control. In this model, during the first phase of needle assembly, the inner rod and needle are assembled at the same time. Completion of the inner rod leads to “substrate switching:” the base stops exporting outer needle proteins and begins to secrete tip proteins and create a mature injectisome. In *Salmonella* the inner rod is composed of a protein called PrgJ, and Marlovits et al. found that overexpressing PrgJ resulted in *shorter* needles, while deleting it resulted in needles that were incredibly long [2], indicating that substrate switching might indeed control the length of needles in this case. Recent work from Hughes and co-workers, however, suggests that the protein InvJ (a homolog of YscP) may serve as a molecular ruler in *Salmonella*, and it is currently unclear which mechanism actually controls the length of *Salmonella* needles [14].

While both the ruler and substrate switching models are qualitatively consistent with various experimental findings, to date there has been no attempt to quantitatively predict how changing crucial parameters in the system (such as the concentration of PrgI, PrgJ or InvJ) would influence the distribution of needle lengths. In this work, we developed a straightforward mathematical model for the substrate switching mechanism in *Salmonella*, which we validated using stochastic simulation. In the case where the majority of needles present on a cell at steady-state are functional (*i.e.* mature), analysis of the model revealed that there is a fixed relationship between the average and variance in needle length. Comparison of our results with the experimental data in Marlovits et al. indicates that a total of around six PrgJ proteins must bind to the inner rod in order to induce substrate switching [2]. The distributions predicted by the model provide excellent agreement with experimental data with only a single free parameter. These findings provide quantitative support for the substrate switching model in *Salmonella*, and also suggest a set of straightforward experiments that would provide stringent tests of the mechanism in future work. Interestingly, the average lengths of needles in *Yersinia* are considerably longer than those observed in *Salmonella*, and the substrate switching model predicts that *Yersinia* needles should have a *much larger* variance in length than has been empirically observed. These results suggest that *Yersinia* and related species may have evolved a different mechanism (namely a ruler protein like YscP) to allow for more precise control over needle length when the needles themselves must be longer. Our work thus provides a mathematical framework for understanding the evolutionary pressures that have shaped length control mechanisms for this critically important virulence factor in bacteria.

## Results

### Ordinary differential equations for the system

**Fig 1:**
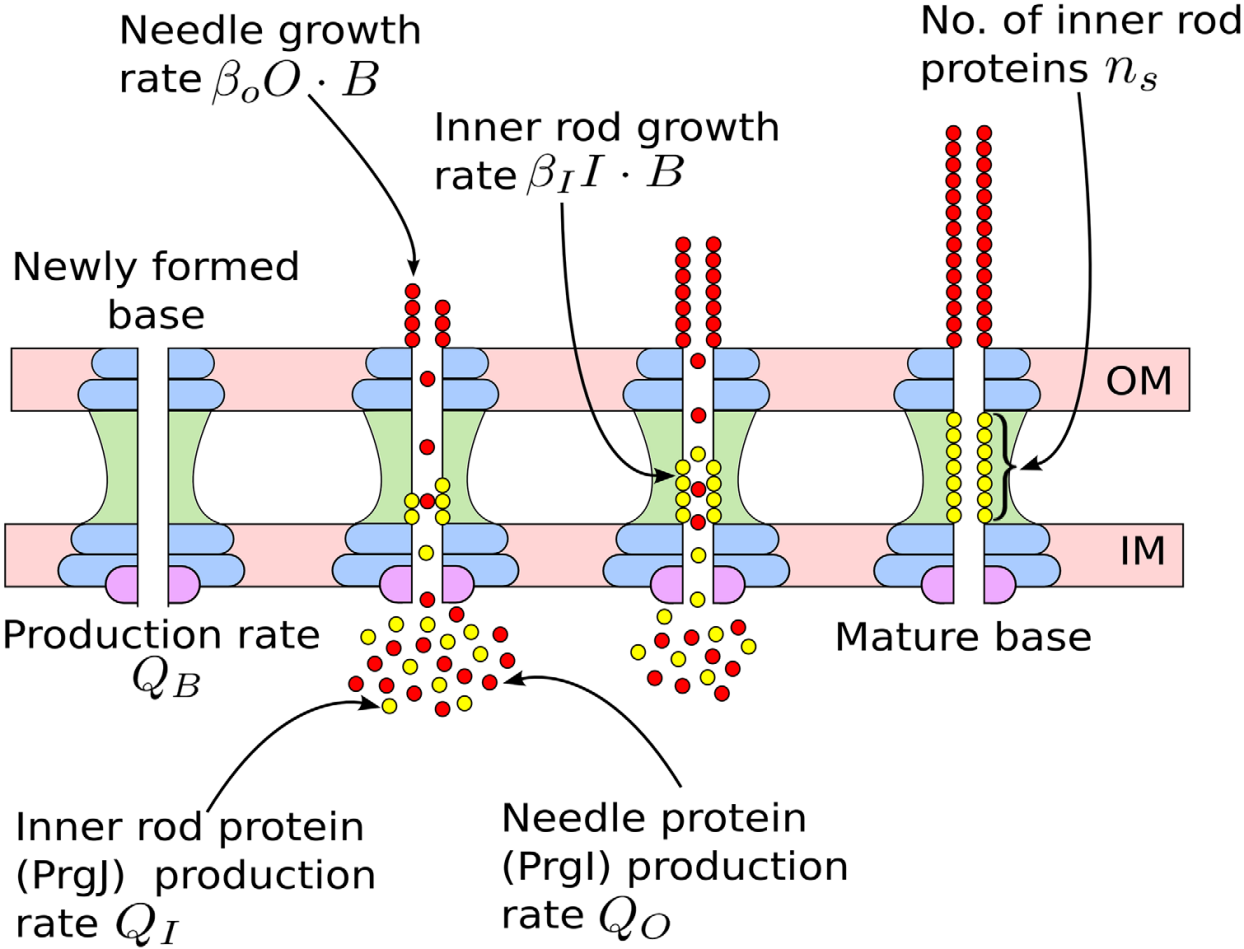
Various stages in the assembly of a needle complex. This schematic shows various stages in a needle complex from base synthesis until maturation. The base spans the inner and the outer membranes (‘IM’ and ‘OM’ respectively) of the bacterial cells. The number of *immature* bases in the cell is represented by the variable *B*, the number of needle proteins by *O* and the inner rod proteins by *I*. Molecules are synthesized or produced at the indicated constant rates *Q* (*Q*_*B*_, *Q*_*O*_, etc.). Binding of needle proteins to bases occurs with rate *β*_*O*_*O* ⋅ *B* and binding of the inner rod proteins to bases occurs at a rate *β*_*I*_*I* ⋅ *B*. A binding event increases the length of the needle (or the inner rod) and reduces the concentration of the relevant protein within the cell. The base reaches maturation when the number inner rod proteins attached reaches a particular value *n*_*s*_, and a mature base can no longer interact with inner rod or needle proteins. The figure does not show the loss of proteins from the cell due to dilution cell division or active degradation. The rates of these processes form the basis of the system of ODEs (eqs (1)–(3)) that allow us to calculate the average values of *B*, *O* and *I* at steady state, as well as our statistical model for the corresponding needle length distribution (eqs (7)–(14)).

To construct a mathematical model of the substrate switching mechanism, we examined the dynamics of the three important constituents of the system, namely the bases, inner rod proteins and needle proteins [1, 2]. We represent the average number of immature bases, inner rod proteins and “outer” needle proteins as *B*, *I* and *O*, respectively. Each of these molecules is synthesized by the cell, and each can be lost from the system due to dilution from cell division or active degradation processes. When a needle (or inner rod) protein binds any given base, this will increase the length of the needle (or inner rod) associated with that base. A schematic of the model is shown in Fig 1. In order to relate the rates of these underlying processes (*i.e.* the *Q*’s and *β*’s in Fig 1) to the distribution of needle lengths one would obtain, we first generated a deterministic system of Ordinary Differential Equations (ODE’s) to calculate the expected average steady-state values of *B*, *I* and *O*. These averages were then used as the basis for a statistical model of the needle length distribution, as described below.

We constructed our ODE model of the dynamics based on the following considerations:

1. **Production:** The individual components in the system are synthesized in the cell at rates specified by parameters *Q*_*B*_, *Q*_*I*_ and *Q*_*O*_. This results in an overall increase in the number of proteins *i.e.* *dI*/*dt* ∼ *Q*_*I*_ (we would have a similar relation for the needle proteins and the bases as well). We assume that the *Q*’s are independent of time. Note that, while the molecular assembly of the base is a complex process in and of itself, in this model we represented the net effect as a constant production of bases.
2. **Degradation:** Dilution from cell division (and any additional active degradation processes) results in effective loss of proteins. Following common convention [19, 20], degradation/dilution is assumed to be first-order and depends on the rates *λ*_*I*_, *λ*_*O*_ and *λ*_*B*_. This results in an overall decrease in the number of proteins *i.e.* *dI*/*dt* ∼ −*λ*_*I*_ *I*. Note that the loss of a base would result in the loss not only of the base complex, but also any inner rod or outer needle proteins associated with it.
3. **Binding:** As mentioned in ref. [2], both the inner rod proteins and the outer needle proteins can bind to an immature base because substrate switching has not occurred yet. This binding is assumed to be a second-order reaction between the proteins and the base; the rate of this reaction is proportional to the product of the number of inner rod proteins (or outer needle proteins) and the number of bases and results in an overall loss in number of proteins: *dI*/*dt* ∼ −*β*_*I*_ ⋅ *I* ⋅ *B*. The binding of an inner rod protein increases the length of the inner rod and the binding of a needle protein increases the length of the needle. Note that we do not explicitly consider the transport process for either inner rod or needle proteins. Also, we do not consider “unproductive” transport events, where, say, a needle protein might dissociate from the tip of the needle before the next needle protein binds. Since including this type of event would simply require a rescaling of the parameters in the model, we neglect these events without loss of generality. We also do not consider the possibility of a dissociated protein re-binging the needle, since the extracellular volume is likely so large that re-binding is highly unlikely (see the Supporting Information for further details).
4. **Substrate switching:** Substrate switching occurs when the number of inner rod proteins inside a base reaches a particular value *n*_*s*_. Once substrate switching occurs, the base is no longer immature, which means that it cannot attach any more inner rod or outer needle proteins. For effective pathogenesis, it is crucial that the base is mature, since this is the only form that can secrete the tip and effector proteins. Note that rate of association of inner rod proteins to an immature base is given by *β*_*I*_ ⋅ *I* ⋅ *B* and for every *n*_*s*_ occurrences of association of inner rod proteins there is one substrate switching event, on average. In our simplified ODE model we thus approximate the maturation rate of bases by *dB*/*dt* ∼ −*β*_*I*_ ⋅ *I* ⋅ *B*/*n*_*s*_

Based on the above considerations, the ordinary differential equations (ODE’s) for the three species is given by:

$$\frac{dO}{dt}=Q_{O}-\lambda_{O}O-\beta_{O}O\cdot B$$ (1)

$$\frac{dI}{dt}=Q_{I}-\lambda_{I}I-\beta_{I}I\cdot B$$ (2)

$$\frac{dB}{dt}=Q_{B}-\lambda_{B}B-\frac{1}{n_{s}}\beta_{I}I\cdot B.$$ (3)

**Fig 2:**
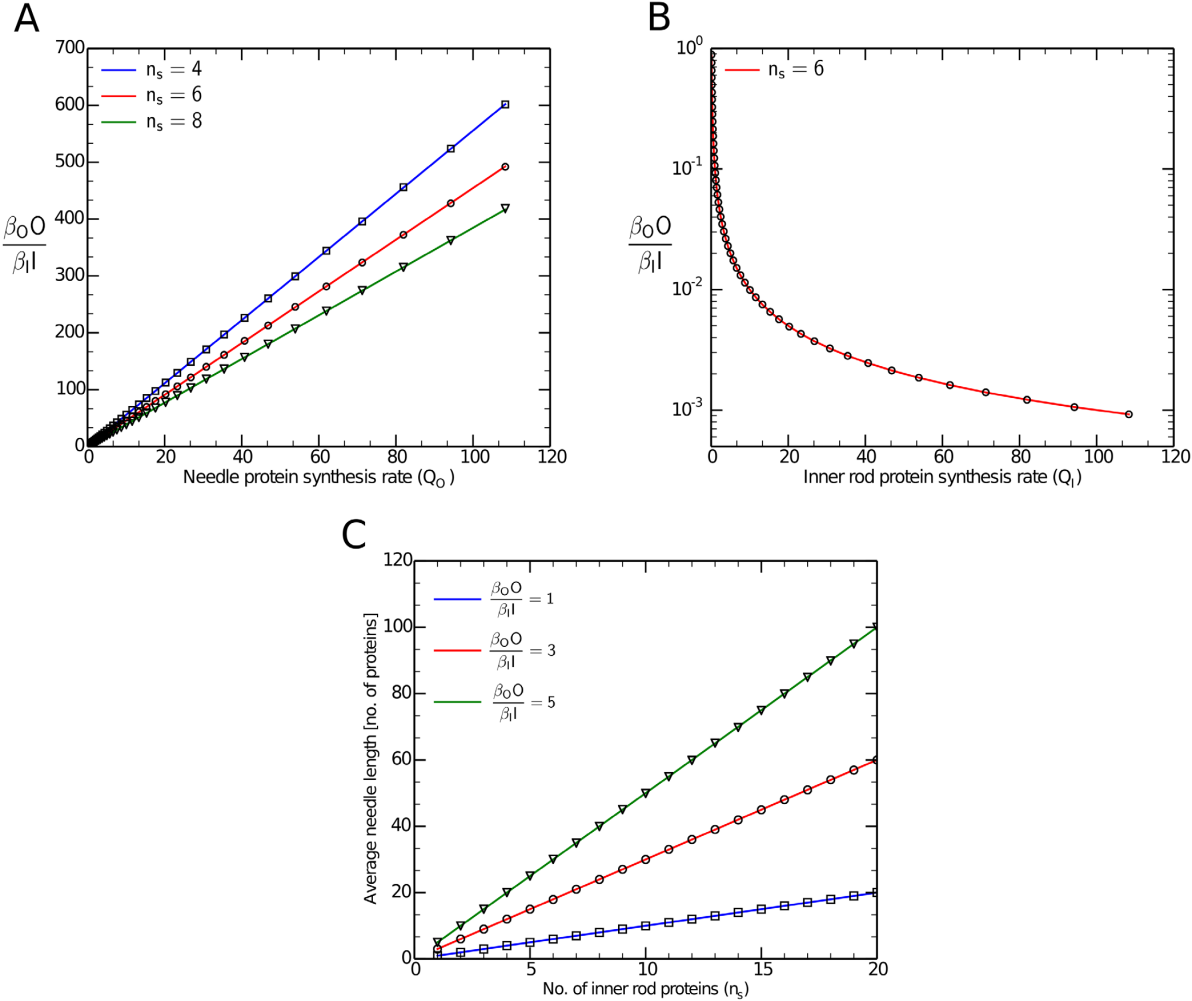
Comparison between theory and simulation. The ratio of the rate parameters for outer needle to that for inner rod proteins controls the average length for a given *n*_*s*_. Figure 2A shows that the ratio *β*_*O*_*O*/*β*_*I*_ *I* increases linearly with the synthesis rate for outer needle proteins *Q*_*O*_, whereas in Figure 2B the ratio has an inverse relation with the synthesis rate for inner rod proteins *Q*_*I*_. The number of inner rod proteins required for substrate switching in Figures 2A and 2B was set to *n*_*s*_ = 6. Figure 2C shows the dependence of the average needle length on *n*_*s*_. In each of the figures, the lines are predictions from the statistical model and the points represent values obtained from stochastic simulation, keeping all other parameters fixed.

The steady-state values of the number of the inner-rod proteins, outer needle proteins and the immature bases can be solved from eqs (1), (2) and (3) as:

$$\bar{I}=\frac{1}{2\beta_{I}\lambda_{I}}\left(\sqrt{C^{2}+4n_{s}\beta_{I}\lambda_{I}\lambda_{B}Q_{I}}-C\right)$$ (4)

$$\bar{O}=\frac{Q_{O}\left(n_{s}\lambda_{B}+\beta_{I}\bar{I}\right)}{n_{s}\beta_{O}Q_{B}+\lambda_{O}\left(n_{s}\lambda_{B}+\beta_{I}\bar{I}\right)}$$ (5)

$$\bar{B}=\frac{n_{s}Q_{B}}{n_{s}\lambda_{B}+\beta_{I}\bar{I}}.$$ (6)

where *C* = *β*_*I*_(*n*_*s*_ *Q*_*B*_ − *Q*_*I*_) + *n*_*s*_ *λ*_*I*_ *λ*_*B*_. While these equations allow us to calculate $\bar{I}$, $\bar{O}$ and $\bar{B}$, we should note that interpreting the above equations in terms of the average number of these proteins in a cell requires that the fluctuations in protein numbers be uncorrelated; that is, 〈*O* ⋅ *B*〉 = 〈*O*〉 ⋅ 〈*B*〉 (and similarly for the interaction of *I* and *B*). As described below, comparison of the predictions from eqs (4)–(6) with the results of stochastic simulations indicate that this assumption holds good when the majority of bases are mature at steady state (see Fig 2). This assumption might well break down in parameter regimes not considered here, however, which would necessitate the consideration of correlated fluctuations in the model. We leave consideration of those regimes to future work.

### Statistical model

To translate the average, “mean field” values for the number of inner rod, outer needle proteins and immature bases from eqs (4)–(6) into the corresponding needle length distribution, we constructed a statistical model of the growth of the inner rod and needle. In this model, it is assumed that the system is already at the steady-state and each of the processes involved (production, binding and degradation) may be treated as uncorrelated sequential random events. For notational convenience we shall omit the bars (*e.g.* $\bar{B}$) and write *I*, *O* and *B* to indicate these steady-state averages below.

Imagine a base is synthesized at time *t* = 0. The probability that this particular base finishes its assembly such that the needle will achieve a length *L* (*i.e.* number of outer needle proteins bound) once it stops growing is:

$$P_{}\left(L\right)=\int_{0}^{\infty }P_{}\left(L|t\right)P_{\text{stop}}\left(t\right)dt,$$ (7)

where *P* (*L*|*t*) is a conditional probability that the needle contains “*L*” proteins at time *t*, and *P*_stop_(*t*) is the probability density that the needle stops growing at time *t*. Note that the rate at which the needle proteins bind to any given immature base is *β*_*O*_*O*, and for a needle of length *L*, the number of consecutive protein bindings required to achieve this length is also *L*. Keeping this in mind and realizing that the process of an outer needle protein binding to an immature base is a Poisson process, we have:

$$P_{}\left(L|t\right)=\frac{{\left(\beta_{O}Ot\right)}^{L}e^{-\beta_{O}Ot}}{L!}.$$ (8)

Note that we take *I* and *O* in this case represent the “mean field” values from eqs (4) and (5), respectively.

To calculate *P*_stop_(*t*), we must consider the two independent mechanisms by which growth of a particular outer needle might cease. Any given base might be degraded *before* its inner rod completes; alternatively, the inner rod might fully assemble (*i.e.* reach *n*_*s*_ proteins) before the base is degraded. The probability density of stopping at time *t* is simply the sum of these probabilities:

$$P_{\text{stop}}\left(t\right)=P_{\text{base}}\left(\text{undeg}|t\right)P_{\text{inner}}\left(\text{complete}|t\right)+P_{\text{base}}\left(\text{deg}|t\right)P_{\text{inner}}\left(\text{incomplete}|t\right),$$ (9)

where *P*_base_(undeg|*t*) and *P*_inner_(incomplete|*t*) denote the cumulative probabilities of the base remaining undegraded and the inner rod remaining incomplete until time *t*, respectively, and *P*_base_(deg|*t*) and *P*_inner_(complete|*t*) denote the instantaneous probability densities that the base degraded and the inner rod is completed at time *t*, respectively. Base degradation occurs at rate *λ*_*B*_, so the probability of the base remaining undegraded until time *t* is *P*_base_(undeg|*t*) = *e*^−*λ*_*B*_*t*^. Similarly, the probability density that the base is degraded precisely at time *t* is *P*_base_(deg|*t*) = *λ*_*B*_ *e*^−*λ*_*B*_*t*^. Once again, the process of each inner rod protein binding to an immature base is a Poisson process with rate *β*_*I*_ *I*. The density of the *waiting times* until the $n_{s}^{\text{th}}$ Poisson event is thus given by an Erlang distribution:

$$P_{\text{inner}}\left(\text{complete}|t\right)=\frac{e^{-\beta_{I}It}{\left(\beta_{I}I\right)}^{n_{s}}t^{n_{s}-1}}{(n_{s}-1)!},$$ (10)

and the probability that the inner rod is incomplete is the cumulative probability that it remains incomplete until time *t*:

$$P_{\text{inner}}\left(\text{incomplete}|t\right)=e^{-\beta_{I}It}\sum_{k=0}^{n_{s}-1}\frac{{\left(\beta_{I}It\right)}^{k}}{k!}.$$ (11)

Using eqs (8)–(11) in Eq (7) and integrating over *t* we obtained an analytical expression for the probability distribution of needle lengths as:

$$P_{}\left(L\right)=\frac{y_{O}^{L}}{L!}\left(\frac{(n_{s}+L-1)!}{(n_{s}-1)!}y_{I}^{n_{s}}+\epsilon \sum_{k=0}^{n_{s}-1}\frac{(k+L)!}{k!}y_{I}^{k+1}\right),$$ (12)

with $y_{O}=\frac{\beta_{O}O}{\beta_{O}O\;+\;\beta_{I}I\;+\;\lambda_{B}}$, $y_{I}=\frac{\beta_{I}I}{\beta_{O}O\;+\;\beta_{I}I\;+\;\lambda_{B}}$ and $\epsilon =\frac{\lambda_{B}}{\beta_{I}I}$.

Interestingly, the last of these dimensionless parameters, *ϵ*, plays a large part in determining the number of bases that are mature at steady state, since it compares the rate of base degradation/dilution (*λ*_*B*_) to the frequency with which any given immature base will bind inner rod proteins (*β*_*I*_ *I*). When *λ*_*B*_ ≫ *β*_*I*_ *I* (equivalently *ϵ* ≫ 1), degradation dominates over inner rod protein binding, and most bases are degraded before they have enough time to bind *n*_*s*_ inner rod proteins. As a result, in this regime, the vast majority of bases are *immature* at steady state. Conversely, when *λ*_*B*_ ≪ *β*_*I*_ *I* (*ϵ* ≪ 1), then inner rod protein binding is much faster than degradation, and the majority of bases are *mature* at steady state. Injectisomes are only functional when they are mature, and since each base complex represents a massive investment of energy in protein synthesis by the cell, we expect that the system has evolved towards a parameter regime where the majority of the bases are mature (and thus functional) at steady state. As such, we take *ϵ* ≪ 1 to be the relevant parameter regime for WT pathogenic bacteria.

Ignoring higher ordered terms in *ϵ* when *ϵ* ≪ 1, the average needle length follows:

$$\langle L\rangle ≃n_{s}\left(\frac{\beta_{O}O}{\beta_{I}I}\right).$$ (13)

Similarly, ignoring higher ordered terms in *ϵ* in the second moment of *L* we obtained the variance in the needle lengths as:

$$\sigma^{2}≃\langle L\rangle +\frac{{\langle L\rangle }^{2}}{n_{s}}.$$ (14)

According to eq (14), the variance in the needle lengths should depend quadratically on the average needle length. Furthermore, given a distribution of needle lengths we can predict the number of inner rod proteins required for substrate switching for any given bacterial species. Using eqs (13) and (14) in eq (12) and ignoring the term in *ϵ* (since *ϵ* ≪ 1), the probability distribution of needle lengths becomes the negative binomial distribution:

$$P_{}\left(L\right)≃\frac{(n_{s}+L-1)!}{L!(n_{s}-1)!}{\left(\frac{\langle L\rangle }{\sigma^{2}}\right)}^{n_{s}}{\left(1-\frac{\langle L\rangle }{\sigma^{2}}\right)}^{L}$$ (15)

**Fig 3:**
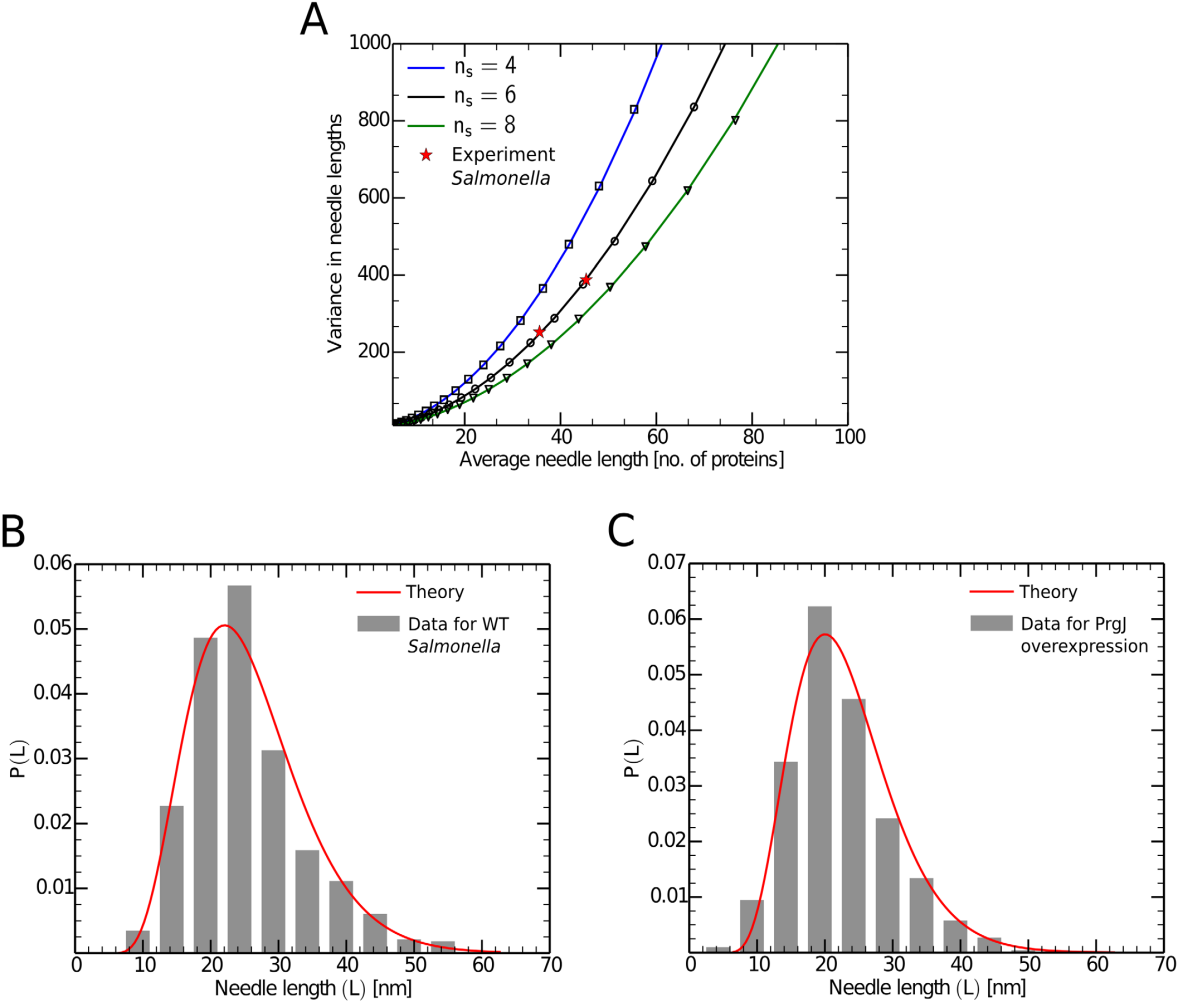
Needle length distributions and comparison with experiment. Figure 3A shows variance in needle lengths versus the average needle lengths. The lines represent predictions from the mathematical model, the points represent results obtained from stochastic simulation for parameter values specified in supplement. The stars are experimental values from data for *Salmonella* in ref. [2]. Figure 3B and 3C are normalized probability distributions of needle lengths for *Salmonella*. Figure 3B is the distribution for “Wild Type” whereas Figure 3C is for a variant with over-expressed inner needle proteins (PrgJ). The histograms represent the experimental values from ref. [2] and the lines represent the values expected according to the statistical model (eq 15), using eq (16) to convert the lengths to nanometers.

The predictions of eqs (13)–(15) are compared against both numerical simulations and experimental predictions in Figs 2 and 3.

### Comparison with stochastic simulation

The results discussed above are a consequence of two very important assumptions about the model: (a) the term *β*_*I*_*I* ⋅ *B*/*n*_*s*_ in eq (3), which approximates the fact that, for every *n*_*s*_ inner rod protein bindings there is one substrate switching event that deletes a base from the pool of immature bases and adds it to the pool of mature bases; (b) the fluctuations in the steady-state values of *I*, *O* and *B* are uncorrelated with each other and would have no effect on the distribution of needle lengths. In order to test the validity of these assumptions, we performed numerical simulations of the assembly process using the Gillespie algorithm [21]. In our simulations the inner rod proteins, outer needle proteins and bases are treated as “agents” that can interact with one another to form needle complexes [22–25]. Each base has an arbitrary number of inner rod and outer needle proteins associated with it, and we track both the pool of mature and immature bases (depending on whether or not substrate switching has occurred for a given base). Note that here each base becomes mature when *precisely* *n*_*s*_ inner rod proteins have associated with it. We also have a pool of free inner rod and outer needle proteins.

The exact value of the parameters in our model have not been determined experimentally, but they can be subjected to a set of reasonable constraints. For instance, the total number of bases associated with a bacterial cell is ∼100, therefore *Q*_*B*_/*λ*_*B*_ ≃ 100. Since “degradation” in this case is assumed to occur largely due to dilution from cell division, the decay parameter *λ* should be of the same for all proteins. Assuming *ϵ* ≪ 1 also constrains the relative values of *λ*_*B*_ and *β*_*I*_. Keeping these constraints in mind, we chose a set of reasonable values (see the Supporting Information) of *Q*′s, *λ*’s and *β*’s, which, along with initial values for the number of bases, inner rod and outer needle proteins, determine the “activities” of synthesis, degradation and binding in our Gillespie simulations. Note that these parameters were chosen simply for comparison between our analytical results and simulations, and were not used for comparisons with experimental data (see below). For every synthesis event, a protein (of the corresponding species) is spontaneously added to the pool of free proteins, whereas in case of a degradation event the protein is deleted from this pool. For binding of an inner rod (or outer needle) protein, a base is chosen randomly and the number of inner rod (or outer needle) proteins associated with it is increased by one. When substrate switching occurs for a particular base, it is deleted from the pool of immature bases and added to the pool of mature bases. For a given set of parameter values, we ran these simulations until the system reached steady-state.

In order to compare our analytical results with the ones obtained from simulation, we focused on the quantity *β*_*O*_*O*/*β*_*I*_ *I*, which, according to eq (13), should control the average needle length. For the chosen set of parameters, we obtained the predicted steady-state values *I*,*O* and *B* using eqs (4)–(6). We examined the the effect of changing the synthesis rate for inner rod and outer needle proteins on *β*_*O*_*O*/*β*_*I*_ *I*, and, as shown in Fig 2A and 2B, the predictions of our model agree with the results obtained from simulation. We also used the predicted steady-state values in eq (13) to obtain the theoretical values of average length and made a similar comparison between theory and simulation examining the effect of changing the number of inner rod proteins required for substrate switching on the average needle lengths (see Fig 2C). As mentioned earlier, our statistical model predicts that the variance in needle lengths should depend quadratically on the average needle length. In order to test this prediction, we varied the synthesis rate of outer needle proteins and obtained average and variance in needle length for each case. This exercise was repeated for different *n*_*s*_ values and as shown in Fig 3A there is very good agreement between the theoretical model and simulation.

### Comparison with experimental data

To further validate our findings, we compared them with experimental results obtained from ref. [2]. In this work, the authors were able to obtain two different distribution of needle lengths in *S. typhimurium*, one for “Wild Type” and the other for a mutant variant where the inner rod proteins (PrgJ) were overexpressed. We calculated the average needle length and the variance in the needle lengths from these distributions. Note that in the experiment, the needle lengths were measured in nanometers (nm), whereas in our work these lengths are represented in number of proteins, and hence we need a transformation that relates the two. As per the structural model of the needle in ref. [7], the height of the first subunit attached to the base is 6.04 nm and the increase in the height of the needle for a every outer needle (PrgI) binding is 0.46 nm, which gives us:

L[innm]=0.46·L[innumberofproteins]+6.04. (16)

We then compared the predictions for average and variances in needle length to those observed for the two experimental distributions (Fig 3A). Interestingly, both of these points lie on the same curve predicted by our model, indicating that the data for *Salmonella* is consistent with six inner rod proteins required for substrate switching (*i.e.* *n*_*s*_ = 6). Substituting *n*_*s*_ = 6 in eq (15), we obtained the analytical probability distribution of needle lengths and compared these with the experimental length distributions from ref. [2] (See Fig 3B and 3C). Note that we obtained excellent agreement between theory and experiment with only one free parameter (*n*_*s*_).

**Fig 4:**
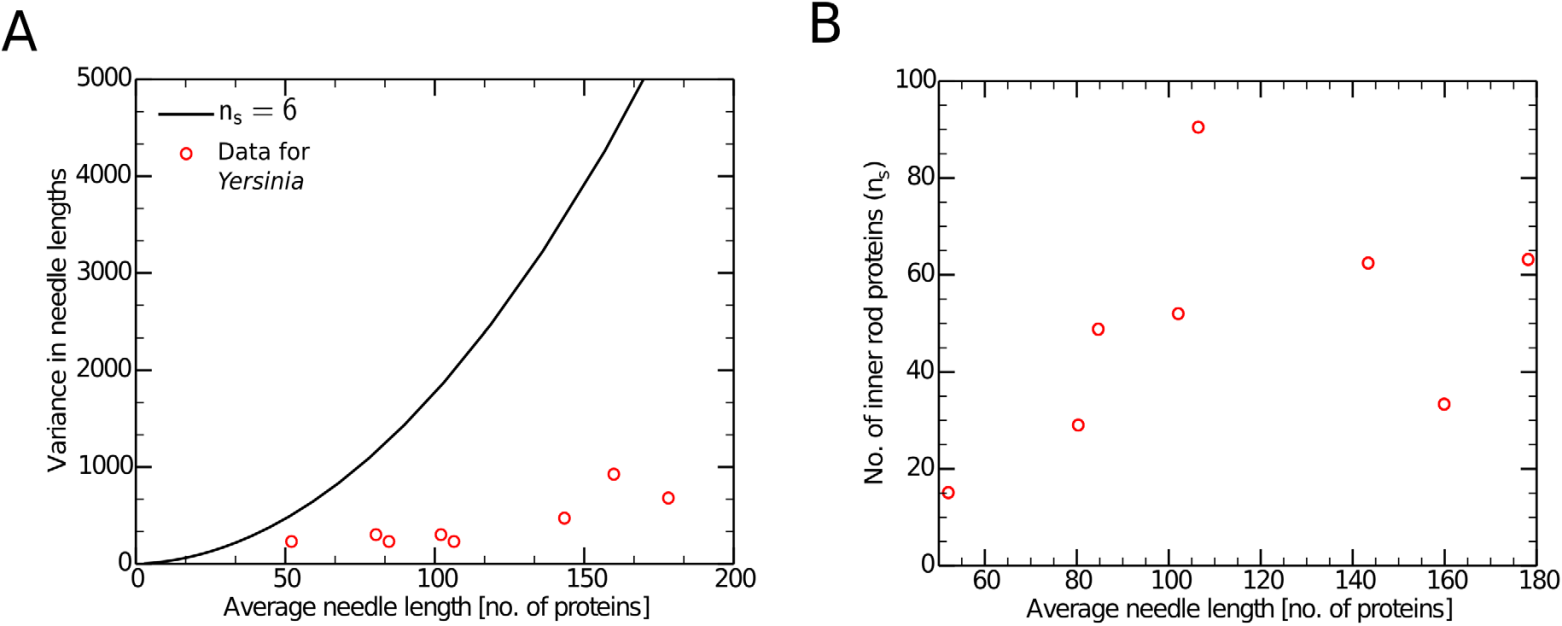
Data from *Yersnia* is not consistent with substrate switching. Figure 4 shows a comparison between the model and experimental data for *Yersinia* taken from ref. [13]. Figure 4A shows that the variance and the average lengths for *Yersinia* needles does not have the expected quadratic relationship for *n*_*s*_ = 6. Figure 4B shows that the number of inner rod proteins required for substrate switching in *Yersinia* is generally not consistent with eq (13), since a wide variety of values of this parameter (ranging between 10 and 100) would be required to explain the data.

As mentioned above, *Y. pestis* appears to have a molecular ruler (YscP) that regulates its needle lengths. The authors of ref. [13] added residues to this ruler protein and obtained needles of different lengths. We observed that the variances in these *Yersinia* needles are much smaller than the predictions for *n*_*s*_ = 6 according to eq (14) (see Fig 4A). Indeed, not only do the variance and average length not satisfy the quadratic relationship with *n*_*s*_ = 6, there is no single *n*_*s*_ value with which the data is consistent. Applying eq (14) to these data, we observe a variety of *n*_*s*_ values scattered between ∼10 to ∼100 (Fig 4B). Unfortunately, we could not obtain data for the full *P*(*L*) distributions for these YscP length mutants, and further tests of the model against this data (*e.g.* comparisons to the distribution in eq (15)) will likely require the collection of new experimental data from *Yersinia*.

## Discussion

Competing models have been proposed to explain how the of the length of the T3SS needle is controlled. While these models are generally considered to be in qualitative agreement with various experimental data sets [1, 2, 13, 14], there has been no attempt to use quantitative approaches to obtain and test predictions from these models in a rigorous way. In this work, we considered the substrate switching mechanism that has been proposed for length control in *Salmonella*, and constructed a mathematical model based on a straightforward interpretation of the interactions thought to be important for this mechanism [1, 2]. The analytical results we obtained from this model are consistent not only with stochastic simulations, but also with available experimental data for *S. typhimurium* [2].

A major prediction of our model (for *ϵ* ≪ 1) is that, for a given population of needles, the variance in the needle lengths should scale *quadratically* with the average needle lengths, parametrized by a consistent value of *n*_*s*_ (*i.e.* the number of inner rod proteins required for substrate switching, see eq 14). This finding implies that the substrate switching mechanism may not be not optimum for bacteria such as *Yersinia pestis*, which need to grow longer needles. If the length of the needles is regulated by substrate switching, larger values of the average length will result in very large variability, potentially impairing pathogenesis [13]. The quadratic relationship between average and variance predicted by our model also suggests a natural set of experiments to provide further evidence for the substrate switching mechanism in *Salmonella* and other bacterial species. One could readily vary the synthesis rate of either PrgI or PrgJ (by using, say, “titratable” promoters whose activity can be controlled by the concentration of an exogenous ligand), measure the average and variance of the resulting needle lengths, and compare those results with the quadratic function predicted by our model (*e.g.* Fig 3 with *n*_*s*_ = 6). Performing such an experiment would provide a very stringent test of the substrate switching mechanism.

Our results also suggest that further structural studies could be helpful in determining the relative feasibility of the substrate switching model. EM structures of the base indicate that it is about 265 Å from top to bottom; the PrgJ portion of the inner rod takes up about 40%, or ∼105 Å, of this distance [3]. If we assume that PrgJ forms a helical structure identical to that of the outer needle, then according to eq (16) it would take ∼9 proteins to span that distance, which is not much larger than the *n*_*s*_ = 6 prediction made by our model. In order to fill that distance with six monomers, the PrgJ portion of the inner rod would have to adopt a somewhat more extended conformation than the needle complex, with ∼0.68 nm/monomer compared to 0.46 nm/monomer. Unfortunately, we currently do not have a higher-resolution structure of PrgJ nor is there a model of the inner rod structure akin to that of the needle [7]. Further structural studies could thus shed light onto whether the *n*_*s*_ = 6 prediction of the model is actually consistent with the number of PrgJ monomers found in the inner rod. Also, it is currently unclear how the information that the inner rod is complete is relayed to the other components of the base complex that ultimately decide which proteins to export and thus drive injectisome maturation. Atomic-resolution structures of more components of this complex will ultimately be necessary in order to understand the allosteric basis of the substrate switching mechanism.

As discussed above, in the alternative ruler protein mechanism, the eponymous ruler (*e.g.* YscP in *Yersinia*) is thought to anchor its C-terminus to the beginning of the needle in the secretion channel and measure the length of the needle; since the secretion channel is very narrow, the ruler will almost certainly block the secretion of other proteins during the time it is bound [5, 7]. This measurement is likely repeated several times during the course of needle growth, until the needle is long enough for the C-terminus of the ruler protein to be in proximity of the needle’s end, which induces needle maturation and the end of needle polymerization [1, 13, 14]. Original support for this model came from studies in *Yersina*, where it was shown that changes in the sequence length of YscP was strongly correlated with the average length of the resulting needles obtained from mutant cells [13]. Interestingly, Wee and Hughes recently provided evidence that changes in the length of the *Salmonella* homolog of YscP, InvJ, also causes concomitant increases in average length [14]. They argued that previous needle-length changes from PrgJ overexpression and deletion mutants in *Salmonella* were the result of changes in the frequency with which the length measurement is made by the ruler protein [2, 14]. Unfortunately, we were unable to extract reliable length distribution data from ref. [14], but inspection of their results indicates it is unlikely that their findings are consistent with a single value of *n*_*s*_, as with the data for YscP mutants in *Yersinia* (Fig 4B) [13, 14].

Developing a full understanding of how parameters like the frequency of length measurement affects the length distribution in the ruler model will require a complete quantitative model similar to substrate switching model described above, which is beyond the scope of the current work. That being said, it is clear that changes in the expression of YscP or other ruler proteins will have distinct effects compared to changing the concentration of the inner rod protein. If the ruler protein is at very high concentration, then the frequency of length checks should also be high, and we would expect that most needles would have lengths quite close to the minimum set by the length of the ruler protein itself, since bases with significantly shorter needles will not mature in that model [1]. As the cocentration of the ruler decreases, however, we would expect some needles to grow too long due to the (relative) decrease in measurement frequency, leading to an asymmetric distribution of needle lengths. In contrast, overexpression of PrgJ or another inner rod protein leads to progressively shorter needles in the substrate switching model, since 〈*L*〉 decreases as 1/*I* (eq (13)). So, while the detailed derivation of *P*(*L*) for the ruler model awaits future study, it is clear that the two models would produce very different distributions based on changes in a similar parameter (namely the concentration of the relevant “length control” protein). In any case, our work emphasizes the role that mathematical models can play in explaining assembly processes and suggesting further experiments that can distinguish between competing mechanisms [19, 26–29].

## Methods

### Numerical simulations

All simulations were performed using a purpose-built simulator that implements the standard Doob-Gillespie approach for the exact stochastic simulation of systems of chemical reactions. Further details on the numerical calculations may be found in the Supporting Information.

## Supporting Information

S1 Text Supplement. This file contains additional information about the mathematical details of the model and details of the stochastic simulation.
(PDF)
