# Supplementary material for: Mathematical Model for Length Control by the Timing of Substrate Switching in the Type III Secretion System
Source: PLoS Comput Biol. 2016 Apr 14;12(4):e1004851. doi: 10.1371/journal.pcbi.1004851 (PMC4831731; doi:10.1371/journal.pcbi.1004851)
Supplement: S1 Text — This file contains additional information about the mathematical details of the model and details of the stochastic simulation. (PDF) [file pcbi.1004851.s001.pdf]

Supporting Information

1 Mathematical derivation for statistical model

1.1 Preliminaries

Notations:

λ_B is the rate of base degradation.

β_I is the association rate of protein I.

β_O is the association rate of protein O.

L is the needle length in terms of number of O proteins.

n_s is the number of I proteins needed for a inner rod to assemble.

The probability that a given needle lives at time t is given by

$$P(t) = \frac{e^{-\lambda_B t} e^{-\beta_I I t} (\beta_I I)^{n_s} t^{n_s - 1}}{(n_s - 1)!} + \lambda_B e^{-\lambda_B t} e^{-\beta_I I t} \sum_{k=0}^{n_s - 1} \frac{(\beta_I I t)^k}{k!} \tag{1}$$

The probability that needle has length L given that it lives at time t is given by

$$P(L|t) = \frac{(\beta_O O t)^L e^{-\beta_O O t}}{L!} \tag{2}$$

Using Equations (1) and (2) we can calculate the probability the a needle has lengh L:

$$\begin{aligned} P(L) &= \int_0^\infty p(L|t) p(t) dt \\ &= \int_0^\infty \frac{(\beta_O O t)^L e^{-\beta_O O t}}{L!} \left[\frac{e^{-\lambda_B t} e^{-\beta_I I t} (\beta_I I)^{n_s} t^{n_s - 1}}{(n_s - 1)!} + \lambda_B e^{-\lambda_B t} e^{-\beta_I I t} \sum_{k=0}^{n_s - 1} \frac{(\beta_I I t)^k}{k!} \right] dt \end{aligned} \tag{3}$$

Let

$$\lambda = \beta_I I + \beta_O O + \lambda_B \tag{4}$$

Eq. (3) can then be written as

$$\begin{aligned} P(L) &= \frac{(\beta_O O)^L}{L!}\left[\frac{(\beta_I I)^{n_s}}{(n_s-1)!}\int_0^\infty t^{n_s+L-1}e^{-\lambda t}dt + \lambda_B\sum_{k=0}^{n_s-1}\frac{(\beta_I I)^k}{k!}\int_0^\infty t^{k+L}e^{-\lambda t}dt\right] \\ &= \frac{(\beta_O O)^L}{L!}\left[\frac{(\beta_I I)^{n_s}}{(n_s-1)!}\frac{\Gamma(n_s+L)}{\lambda^{n_s+L}} + \lambda_B\sum_{k=0}^{n_s-1}\frac{(\beta_I I)^k}{k!}\frac{\Gamma(k+L+1)}{\lambda^{k+L+1}}\right] \\ &= \frac{(\beta_O O)^L}{L!}\left[\frac{(\beta_I I)^{n_s}}{(n_s-1)!}\frac{(n_s+L-1)!}{\lambda^{n_s+L}} + \lambda_B\sum_{k=0}^{n_s-1}\frac{(\beta_I I)^k}{k!}\frac{(k+L)!}{\lambda^{k+L+1}}\right] \\ &= \frac{y_O^L}{L!}\left[\frac{(n_s+L-1)!}{(n_s-1)!}\,y_I^{n_s} + \delta\sum_{k=0}^{n_s-1}\frac{(k+L)!}{k!}\,y_I^k\right] \end{aligned} \tag{5}$$

where

$$y_O = \frac{\beta_O O}{\lambda}\,, \qquad y_I = \frac{\beta_I I}{\lambda}\,, \qquad \delta = \frac{\lambda_B}{\lambda}$$

and n_s and L are integers. Note that the normalization condition of $P(L)$ is good,

$$\begin{aligned} \sum_{L=0}^{\infty} P(L) &= \sum_{L=0}^{\infty}\frac{y_O^L}{L!}\left[\frac{(n_s+L-1)!}{(n_s-1)!}\,y_I^{n_s} + \delta\sum_{k=0}^{n_s-1}\frac{(k+L)!}{k!}\,y_I^k\right] \\ &= \frac{y_I^{n_s}}{(n_s-1)!}\sum_{L=0}^{\infty}\frac{(n_s+L-1)!}{L!}y_O^L + \delta\sum_{k=0}^{n_s-1}\frac{y_I^k}{k!}\sum_{L=0}^{\infty}\frac{(k+L)!}{L!}y_O^L \\ &= \frac{y_I^{n_s}}{(1-y_O)^{n_s}} + \delta\sum_{k=0}^{n-1}\frac{y_I^k}{(1-y_O)^{k+1}} \;=\; z_I^n + (1-z_I)\sum_{k=0}^{n-1} z_I^k \;=\; 1 \end{aligned} \tag{6}$$

where

$$z_I = \frac{y_I}{1-y_O} = \frac{\beta_I I}{\beta_I I + \lambda_B}\,, \qquad z_O = \frac{y_O}{1-y_O} = \frac{\beta_O O}{\beta_I I + \lambda_B}\,, \qquad \frac{\delta}{1-y_O} = 1 - z_I$$

and for the summation over L we have used

$$\frac{1}{(1-y)^{m+1}} = \frac{1}{m!}\sum_{l=0}^{\infty}\frac{(m+l)!}{l!}y^l$$

1.2 Calculation of average needle length

$$
\begin{aligned}
\langle L\rangle &= \sum_{L=0}^{\infty} L P(L) \\
&= \sum_{L=1}^{\infty} \frac{y_O^L}{(L-1)!}\left[\frac{(n+L-1)!}{(n-1)!}\, y_I^{n_s} + \delta \sum_{k=0}^{n_s-1} \frac{(k+L)!}{k!}\, y_I^k\right] \\
&= \frac{y_I^{n_s}}{(n_s-1)!}\sum_{L=1}^{\infty}\frac{(n_s+L-1)!}{(L-1)!} y_O^L + \delta \sum_{k=0}^{n_s-1}\frac{y_I^k}{k!}\sum_{L=1}^{\infty}\frac{(k+L)!}{(L-1)!} y_O^L \\
&= \frac{y_I^{n_s}}{(n_s-1)!}\sum_{l=0}^{\infty}\frac{(n_s+l)!}{l!} y_O^{l+1} + \delta \sum_{k=0}^{n_s-1}\frac{y_I^k}{k!}\sum_{l=0}^{\infty}\frac{(k+l+1)!}{l!} y_O^{l+1} \\
&= y_O\left[\frac{n_s y_I^{n_s}}{(1-y_O)^{n_s+1}} + \delta\sum_{k=0}^{n_s-1}\frac{(k+1)y_I^k}{(1-y_O)^{k+2}}\right] \;=\; y_O\left[\frac{n_s y_I^{n_s}}{(1-y_O)^{n_s+1}} + \delta\sum_{k=1}^{n_s}\frac{k y_I^{k-1}}{(1-y_O)^{k+1}}\right] \\
&= \frac{y_O}{1-y_O}\left[n_s z_I^{n_s} + \frac{\delta}{1-y_O}\sum_{k=1}^{n_s} k z_I^{k-1}\right] \\
&= z_O\left[n_s z_I^{n_s} + (1-z_I)\frac{d}{dz_I}\sum_{k=1}^{n_s} z_I^k\right] \\
&= z_O\frac{1-z_I^{n_s}}{1-z_I}
\end{aligned} \tag{7}
$$

Let

$$\epsilon = \frac{\lambda_B}{\beta_I I}$$

Then

$$z_I = \frac{1}{1+\epsilon}, \qquad \frac{z_O}{1-z_I} = \frac{\beta_O O}{\beta_I I}\epsilon^{-1}$$

and

$$\langle L\rangle = \left(\frac{\beta_O O}{\beta_I I}\right)\frac{(1+\epsilon)^{n_s}-1}{\epsilon(1+\epsilon)^{n_s}} \tag{8}$$

(a) For $\epsilon \ll 1$ or $\beta_I I \gg \lambda_B$,

$$\langle L\rangle \simeq n_s\left(\frac{\beta_O O}{\beta_I I}\right) \tag{9}$$

For $\epsilon \ll 1$, therefore, $\langle L\rangle$ increases linearly with $(\beta_O O/\beta_I I)$ and the slope can be used to determine n experimentally.

(b) For $\epsilon \gg 1$ or $\beta_I I \ll \lambda_B$,

$$\langle L\rangle \simeq \frac{1}{\epsilon}\left(\frac{\beta_O O}{\beta_I I}\right) = \frac{1}{\lambda_B}(\beta_O O) \tag{10}$$

In this case, $\langle L \rangle$ is independent of n and $\beta_I I$.

1.3 Calculation of the variance

To calculate the standard deviation of L, we notice that

$$\left\langle L^2 \right\rangle = \sum_{L=0}^{\infty} L^2 P(L) = \sum_{L=0}^{\infty} L(L-1+1)P(L) = \langle L \rangle + \sum_{L=0}^{\infty} L(L-1)P(L) \tag{11}$$

and

$$\sum_{L=0}^{\infty} L(L-1)P(L) = \frac{y_I^n}{(n-1)!} \sum_{L=2}^{\infty} \frac{(n+L-1)!}{(L-2)!}\, y_O^L + \delta \sum_{k=0}^{n-1} \frac{y_I^k}{k!} \sum_{L=2}^{\infty} \frac{(k+L)!}{(L-2)!}\, y_O^L \tag{12}$$

Since

$$\sum_{L=2}^{\infty} \frac{(k+L)!}{(L-2)!}\, y_O^L = \sum_{l=0}^{\infty} \frac{(k+l+2)!}{l!}\, y_O^{l+2} = (k+2)! \frac{y_O^2}{(1-y_0)^{k+3}} \tag{13}$$

$$\begin{aligned}
\sum_{L=0}^{\infty} L(L-1)P(L) &= \frac{y_I^{n_s}}{(n_s-1)!} \frac{(n_s+1)!\, y_O^2}{(1-y_0)^{n_s+2}} + \delta \sum_{k=0}^{n-1} \frac{y_I^k}{k!} \frac{(k+2)!\, y_O^2}{(1-y_0)^{k+3}} \\
&= \left(\frac{y_O}{1-y_O} \right)^2 \left[n_s(n_s+1) \frac{y_I^{n_s}}{(1-y_O)^{n_s}} + \frac{\delta}{1-y_O} \sum_{k=0}^{n_s-1} (k+2)(k+1) \frac{y_I^k}{(1-y_O)^k} \right] \\
&= z_O^2 \left[n_s(n_s+1) z_I^{n_s} + (1-z_I) \sum_{k=0}^{n_s-1} (k+2)(k+1) z_I^k \right] \\
&= z_O^2 \left[n_s(n_s+1) z_I^{n_s} + (1-z_I) \frac{d^2}{dz_I^2} \sum_{k=0}^{n_s-1} z_I^{k+2} \right] \\
&= z_O^2 \left[n_s(n_s+1) z_I^{n_s} + (1-z_I) \frac{d^2}{dz_I^2} \left(\frac{z_I^2 - z_I^{n_s+2}}{1-z_I} \right) \right] \\
&= 2 z_O^2 \frac{1-(n_s+1) z_I^{n_s} + n_s z_I^{n_s+1}}{(1-z_I)^2}
\end{aligned} \tag{14}$$

From Eq. (7),

$$z_I^n = \frac{z_O - (1-z_I)\langle L \rangle}{z_O}$$

and

$$\begin{aligned}
\sum_{L=0}^{\infty} L(L-1)P(L) &= 2 z_O \left[\left(n_s + \frac{1}{1-z_I} \right) \langle L \rangle - n_s \frac{z_O}{1-z_I} \right] \\
&= \frac{2}{1+\epsilon} \left(\frac{\beta_O O}{\beta_I I} \right) \left[\left(n_s + 1 + \frac{1}{\epsilon} \right) \langle L \rangle - \frac{n_s}{\epsilon} \left(\frac{\beta_O O}{\beta_I I} \right) \right]
\end{aligned} \tag{15}$$

Therefore

$$\begin{aligned}\left\langle L^2\right\rangle &= \langle L\rangle + \sum_{L=0}^{\infty} L(L-1)P(L)\\ &= \langle L\rangle + \frac{2}{1+\epsilon}\left(\frac{\beta_O O}{\beta_I I}\right)\left[\left(n_s+1+\frac{1}{\epsilon}\right)\langle L\rangle - \frac{n_s}{\epsilon}\left(\frac{\beta_O O}{\beta_I I}\right)\right]\end{aligned} \tag{16}$$

and

$$\left\langle L^2\right\rangle - \langle L\rangle^2 = \langle L\rangle + \frac{2}{1+\epsilon}\left(\frac{\beta_O O}{\beta_I I}\right)\left[\left(n_s+1+\frac{1}{\epsilon}\right)\langle L\rangle - \frac{n_s}{\epsilon}\left(\frac{\beta_O O}{\beta_I I}\right)\right] - \langle L\rangle^2 \tag{17}$$

(a) For $\epsilon \ll 1$,

Substituting Eq. (8) into Eq. (17) and keeping only the zeroth-order term of ϵ yields

$$\left\langle L^2\right\rangle - \langle L\rangle^2 \simeq \langle L\rangle + n_s\left(\frac{\beta_O O}{\beta_I I}\right)^2 \simeq \langle L\rangle + \frac{1}{n_s}\langle L\rangle^2 \tag{18}$$

(b) For $\epsilon \gg 1$,

Substituting Eq. (8) into Eq. (17) and keeping only the lowest order term of $1/\epsilon$ yields

$$\left\langle L^2\right\rangle - \langle L\rangle^2 \simeq \langle L\rangle + \frac{1}{\epsilon^2}\left(\frac{\beta_O O}{\beta_I I}\right)^2 \simeq \langle L\rangle + \langle L\rangle^2 \tag{19}$$

1.4 Distribution of the Needle Length at $\epsilon \ll 1$

Let

$$M_1 = \langle L\rangle \qquad \text{and} \qquad M_2 = \left\langle L^2\right\rangle - \langle L\rangle^2$$

From Eqs. (9) and (18),

$$n_s = \frac{M_1^2}{M_2 - M_1}\,, \qquad \frac{\beta_O O}{\beta_I I} = \frac{M_2}{M_1} - 1$$

and

$$\begin{aligned}y_I &= \frac{1}{1+\epsilon+(\beta_O O/\beta_I I)} = \frac{M_1}{M_2+\epsilon M_1} \simeq \frac{M_1}{M_2}\\ y_O &= \frac{(\beta_O O/\beta_I I)}{1+\epsilon+(\beta_O O/\beta_I I)} = \frac{M_2-M_1}{M_2+\epsilon M_1} \simeq 1-\frac{M_1}{M_2}\end{aligned}$$

At $\epsilon \ll 1$

$$\begin{aligned} P(L) &= \frac{y_O^L}{L!}\left[\frac{(n_s+L-1)!}{(n_s-1)!}\,y_I^{n_s} + \delta\sum_{k=0}^{n-1}\frac{(k+L)!}{k!}\,y_I^k\right] \\ &= \frac{(n_s+L-1)!}{L!(n-1)!}\,y_I^{n_s}y_O^L \\ &= \frac{(n_s+L-1)!}{L!(n-1)!}\left(\frac{M_1}{M_2}\right)^{n_s}\left(1-\frac{M_1}{M_2}\right)^L \end{aligned} \tag{20}$$

where

$$n_s = \frac{M_1^2}{M_2 - M_1} \tag{21}$$

2 Details of the stochastic simulation

We treated the bases as individual, discrete "agents" in our simulations. There are two integers associated with each base; the first represents the number of inner rod proteins, and can take values from 0 to n_s. The second represents the number of needle proteins associated with that particular base, and can take any positive integer value. We maintained two separate populations of bases; the set of "immature" bases, with less than n_s inner rod proteins (call this set B), and "mature" bases, with exactly n_s inner rod proteins (call this set B'). The cardinality of these sets (i.e. the number of mature and immature bases) are represented as B and B', respectively, with the total number of bases $B_{\text{tot}} = B + B'$ (see Fig. ??). We used two additional integers, I and O, to track the total number of inner rod and needle proteins.

The possible chemical reactions in this system, and their influences on the values of the variables described above, are summarized in Fig. ?? and are based on the model described in the main text. Note that we initialize the simulation with a set of completely immature bases that each have 0 inner rod and outer needle proteins. When a new base is synthesized during the simulation, we created a new immature base with 0 inner and outer proteins, and added this base to the immature pool. When a base is degraded, we chose one base at random from the set of total bases and removed it from the simulation; all bases, regardless of whether or not they are mature, have an equal probability of being chosen for degradation.

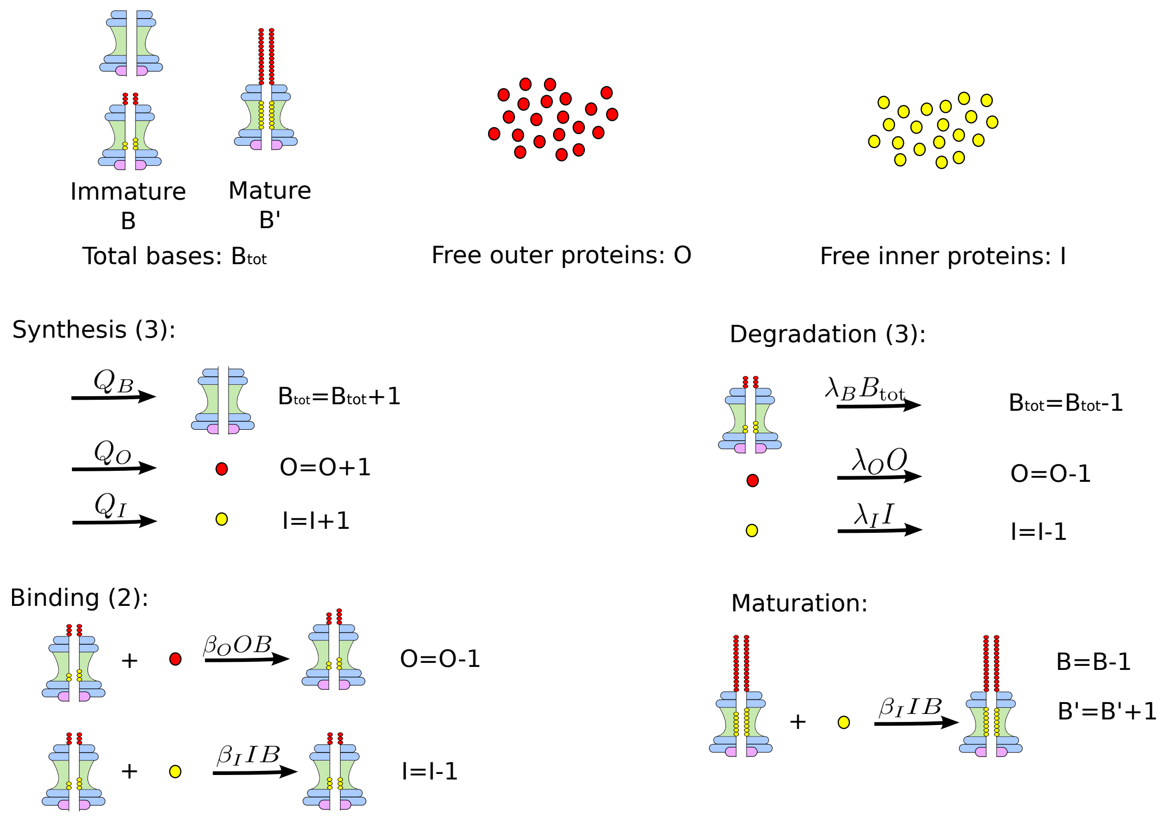

Figure 1: Figure shows processes incorporated in the simulation. The top row shows the pool of immature bases, mature bases, free needle proteins and free inner rod proteins. The middle row shows 3 types of synthesis and degradation events that can occur. The bottom row shows the 2 types of binding events and a binding event that results in maturation.

Only immature bases can participate in binding reactions with inner rod or needle proteins, and when any given base binds to its "last" inner-rod protein (i.e. binds to an inner-rod protein and undergoes the transition from $n_s - 1$ to n_s inner rod proteins), that base becomes mature and is moved from the immature to mature pool.

A binding event in our model always results in an increase in the needle (or inner-rod) protein number associated with the base. As a result, we ignore a number of possible scenarios that might occur during needle assembly. For instance, a needle protein might dissociate from the needle; since the needle itself is a helix, the only protein that is likely to unbind is

the one at the very tip. Alternatively, a needle protein might be exported by the base but simply never attach to the growing needle. Both of these scenarios represent "unsuccessful export," in that the export event would reduce the number of needle proteins within the cell without a net increase in the needle length. This would mean that the $\beta_O O$ term in our deterministic equations (equations (1)-(3) in the main text) would no longer correspond to the $\beta_O O$ term in our statistical model, since the frequency of needle protein export would no longer be equal to the frequency of needle growth. We could thus define a separate rate constant for the statistical model, $\beta'_O < \beta_O$, to account for the fraction of export events that are unsuccessful. Since this would simply change the numerical relationship between the predictions of our deterministic model and the input to our statistical model, and would have no impact on the overall behavior of the system. As such, we neglect unsuccessful export events without a loss of generality.

Unsuccessful export could also lead to the accumulation of needle protein monomers in the extracellular space, which could bind to needles and extend them through a mechanism other than export. In our model, however, we assume that the extracellular volume is much larger than the intracellular volume: as a result, re-binding events are likely to be very rare and are also neglected. In any case, re-binding would simply change the relative values of β'_O and β_O, so consideration of this effect would also not impact our results.

We implemented this simulation using the standard "Gillespie-Doob" approach for exact simulation of stochastic chemical kinetics (1). In this case, the "propensity" or "activity" of any given reaction was calculated according to the functions over the arrows in Fig. **??**. The rate constants in this case have exactly the same definitions as they do for the deterministic model described in the main text. The values for the parameters (i.e. Q's, β's, etc.) used in this study are summarized in Table 1. All simulations were run until they achieved steady state; as set of representative dynamics for various variables as they approach steady state are shown in Fig. **??**.

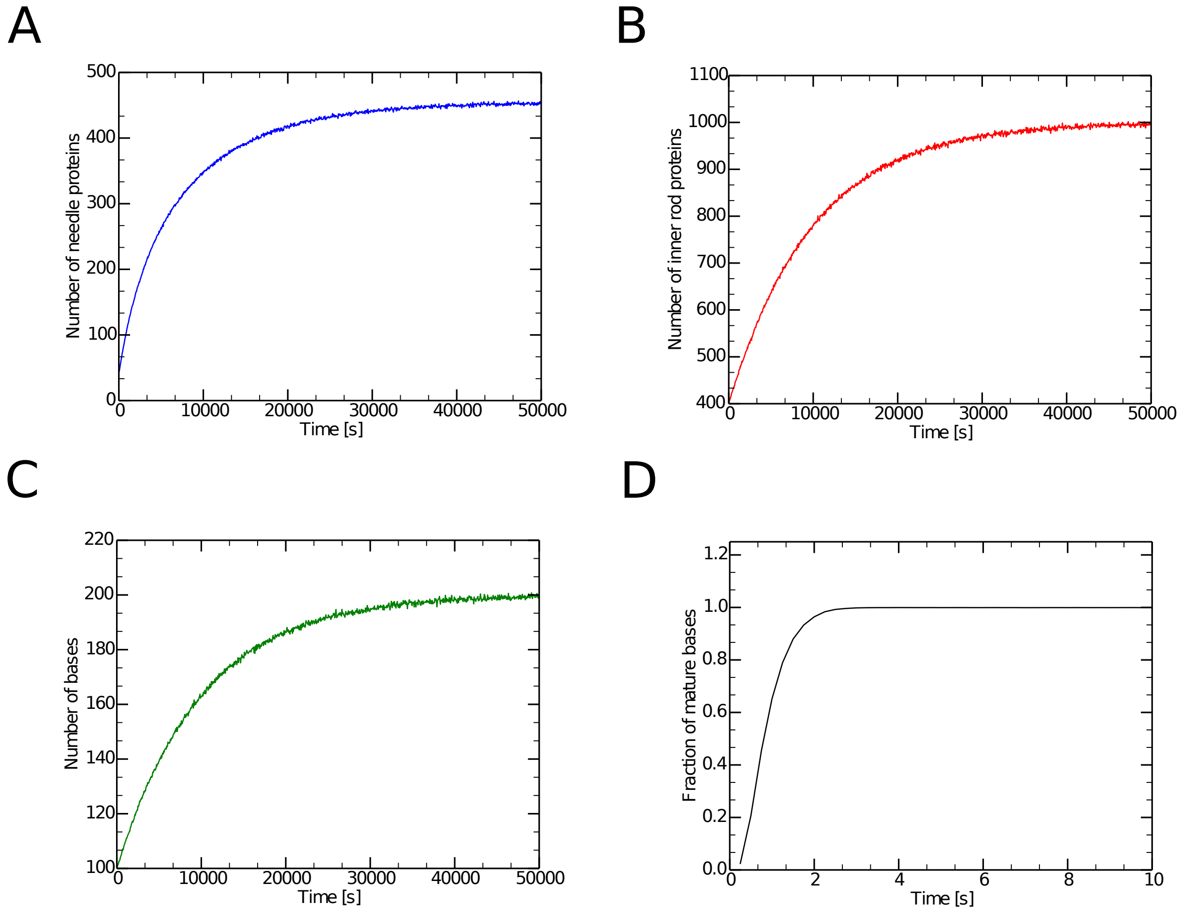

Figure 2: Figure shows the saturation of A: the number needle protein B: the number of inner rod proteins C: the number of bases and D: the fraction of mature bases, as time progresses in the stochastic simulation for the parameter values in Table 1.

Table 1: Parameter values used in simulation

Parameter	Value
Q_B	0.1 [molecules sec^{-1}]
Q_I, Q_O	$0.1-100$ [molecules sec^{-1}]
λ_B, λ_I, λ_O	5×10^{-4} [sec^{-1}]
β_I, β_O	10^{-2} [$\text{molecules}^{-1}\text{sec}^{-1}$]
n_s	4-20

Note: Q_I and Q_O were varied logarithmically from 0.1 to 100 [molecules sec^{-1}]

References

[1] Gillespie D T (1977) Exact stochastic simulation of coupled chemical reactions *J Phys Chem* 81:2340-2361.
